# Genome‐wide selective detection of Mile red‐bone goat using next‐generation sequencing technology

**DOI:** 10.1002/ece3.8165

**Published:** 2021-10-12

**Authors:** Yong‐Meng He, Qiong‐Hua Hong, Dong‐Ke Zhou, Shi‐Zhi Wang, Bai‐Gao Yang, Ying Yuan, Wei‐Yi Zhang, Yong‐Fu Huang, Guang‐Xin E

**Affiliations:** ^1^ Chongqing Key Laboratory of Forage & Herbivore College of Animal Science and Technology Chongqing Engineering Research Centre for Herbivores Resource Protection and Utilization Southwest University Chongqing China; ^2^ Yunnan Animal Science and Veterinary Institute Kunming China

**Keywords:** CNV, genome‐wide sequence, goat, red‐bone, SNP

## Abstract

The ecotype population of goats (*Capra hircus*) was created by long‐term artificial selection and natural adaptation. Mile red‐bone goat is an indigenous breed with visible red bones, and its special bone structure has received extensive attention. This study aimed to identify genetic variants and candidate genes associated with specific bone phenotypes using next‐generation sequencing technology (NGS). The results revealed that 31,828,206 single nucleotide polymorphisms (SNPs) were obtained from 72 goats (20 Mile red‐bone goats and 52 common goats) by NGS. A total of 100 candidate genes were identified on the basis top 1% window interaction from nucleotide diversity (*π*), *π* ratio (*π*_A_/*π*_B_), and pairwise fixation index (*F*_ST_). Exactly 77 known signaling pathways were enriched. Specifically, three coding genes (*NMNAT2*, *LOC102172983*, and *PNLIP*) were annotated in the vitamin metabolism signaling pathways, and *NCF2* was annotated to the osteoclast (OC) differentiation pathway. Furthermore, 5862 reliable copy number variations (CNVs) were obtained, and 14 and 24 genes were annotated with the top 1‰ CNV based on *F*_ST_ (>0.490) and *V*_ST_ (>0.527), respectively. Several pathways related to bone development and metabolism of exogenous substances in vivo, including calcium signaling pathway, OC differentiation, and glycerophospholipid metabolism, were annotated. Specifically, six genes from 19 candidate CNVs, which were obtained by interaction of the top 1‰ CNVs with *F*_ST_ and *V*_ST_, were annotated to mucin‐type O‐glycan biosynthesis and metabolic pathways. Briefly, the results implied that pseudopurpurin and specific genetic variants work together to contribute to the red‐bone color and specific bone structure of Mile red‐bone goat. This study is helpful to understanding the genetic basis of the unique bone phenotype of Mile red‐bone goats.

## Graphical abstract

Mile red‐bone goat is an indigenous breed with visible red bones and special bone structure has received extensive attention. The aim of this study was to identify genetic variants and candidate genes associated with specific bone phenotypes using next‐generation sequence technology (NGS). This study is helpful to understanding the genetic basis of unique bone phenotype of Mile red‐bone goats.

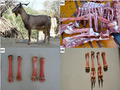

## 1 INTRODUCTION

**FIGURE 1:**
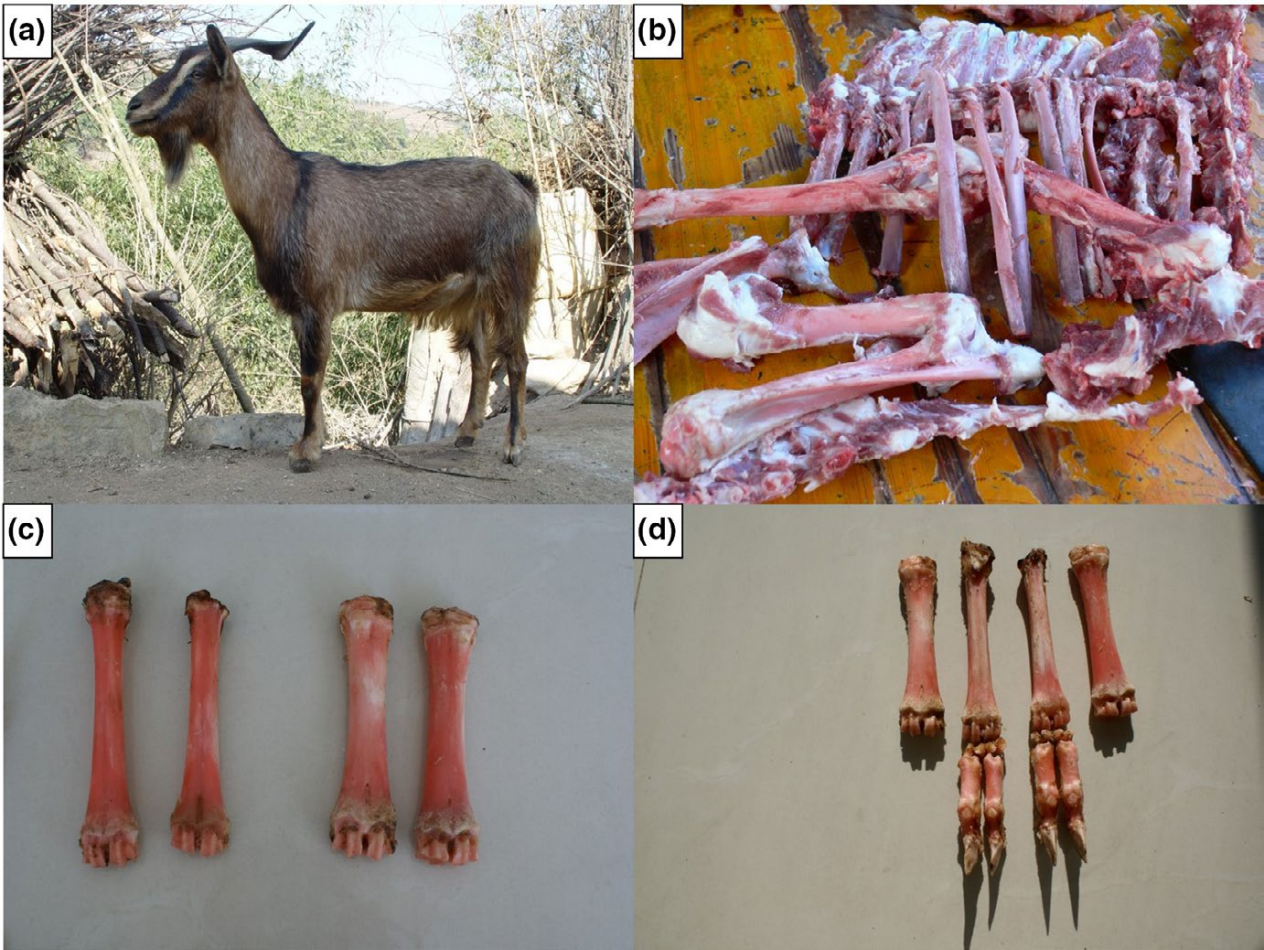
Appearance of the Mile red‐bone goat and its red‐bone color. (a) Red‐bone goat; (b) trunk bone of red‐bone goat; (c–d) bone from the four limbs of Mile red‐bone goat

Mile red‐bone goat (*Capra hircus*) is a rare and unique native goat breed that is mainly distributed in Mile County of Yunnan Province. It has a firm body and yellowish brown or dark brown coat color, and its bones have a significant visible red color (Figure 1) (China National Commission of Animal Genetic Resources, 2011). Pseudopurpurin is the material responsible for the red‐bone color and is the main component of *Rubia cordifolia* L., one of the plants eaten by Mile red‐bone goats (Usai & Marchetti, 2010; Wu, Li, Han, Li, Liu, et al., 2012; Wu, Li, Han, Li, Wang, et al., 2012). *Ru* *bia* has more than 60 species in the world, and it is mainly distributed in Europe, Asia, South Africa, and America (Hua, 2008). *Rubia cordifolia* L. is used not only as a popular feed for herbivores but also as a coloring agent in the textile industry (LaBerge, 2018).

As an anthraquinone compound, pseudopurpurin is used in a wide range of industrial dyestuff (Shahid et al., 2019). Several studies have shown the effects of pseudopurpurin on animal bone mineral density and bone geometry (Wu, Li, Han, Li, Liu, et al., 2012; Wu, Li, Han, Li, Wang, et al., 2012) and its selective cytotoxicity, adhesion, and migration regulation of melanoma cell lines (A2058 and HT168‐M1) (Lajkó et al., 2015). Numerous anthraquinone compounds are used as industrial dyes (Fleischmann et al., 2015) and involved in the inhibition of bone resorption of osteoclasts (OCs) and bone injuries (He et al., 2019).

However, the reason why the corresponding special physiology phenotype, that is, red‐bone color, is only observed in Mile red‐bone goats remains incompletely understood. *Rubia* is a widely distributed food source for herbivores worldwide, and herbivores ingest a large amount of pseudopurpurin and other anthraquinone compounds.

Valuable molecular markers with economic traits and phenotypes of goat (E et al., 2019; Zhang et al., 2019) had been identified through whole‐genome sequencing technology, and their corresponding underlying genetic mechanism can be explained. Therefore, in this study, we investigated genetic variants and candidate genes related to the red‐bone color and extremely high bone density of Mile red‐bone goats using next‐generation sequencing technology (NGS), which can be helpful to further understanding of their hereditary basis.

## 2 MATERIALS AND METHODS

The experimental conditions of this study were approved by the Committee on the Ethics of Animal Experiments of Southwest University (No. [2007] 3) and the Animal Protection Law of China. One milliliter of venous blood was collected from each individual (20 Mile red‐bone goats) and stored in a refrigerator at −80℃. The genomic DNA was extracted with a Tiangen DNA extraction kit (Tiangen Biotech Co., Ltd.). Then, the DNA of 20 Mile red‐bone goat individuals (case group, HG) was subjected to genome‐wide sequencing using the BGISEQ‐500 platform (Beijing Genomics Institute, China) with >20‐fold genome sequences (SRR11696580‐99). The control group (CG, common goats) comprised 52 goats from two units. One included 13 Chinese local goats with more than 10‐fold genome re‐sequences from our other parallel project with the Illumina HiSeq ×10 PE150™ platform, and the other was downloaded from known public data.

All high‐quality clean reads (HQR) of all animals were mapped to the goat reference genome (ARS01) after filtering out the adapter and low‐quality raw paired reads with BWA 0.7.17‐r1188 (E et al., 2019). Single nucleotide polymorphisms (SNPs) were called and classified for each individual by using SAMtools and ANNOVAR, respectively. Selective sweep regions were estimated depending on the interception of three different parameters, namely, nucleotide diversity (*π*) (Nei & Li, 1979), *π* ratio (*π*_A_/*π*_B_), and pairwise fixation index (*F*_ST_) (Hudson et al., 1992). A 40‐kb sliding chromosome window (CW) approach with a 20‐kb step size was applied to calculate these parameters with PopGenome (Pfeifer et al., 2014), and all related graphs were drawn by R scripts.

CNVcaller software (Wang et al., 2017) was used to detect and calculate the genome‐wide copy number variation (CNV) with reads duplication (RD) < 2 of the deletion area and RD > 2 of the duplication area (Wang et al., 2017). In addition, the reliable CNV was obtained for subsequent research based on silhouette scores and minimum allele frequencies greater than 0.05. The *F*_ST_ (Hudson et al., 1992) and *V*_ST_ parameters were used to measure the difference in the size of each CNV between groups. *V*_ST_ was calculated as follows: (*V*_total_ − (*V*_pop1_ × *N*_pop1_ + *V*_pop2_ × *N*_pop2_)/*N*_total_)/*V*_total_, where *V*_total_ is the total variance, *V*_pop_ is the CN variance for each respective population, *N*_pop_ is the sample size for each respective population, and *N*_total_ is the total sample size. The value of *V*_ST_ is between 0 and 1, and a large value means a great difference in the CNV between regions in the population (Redon et al., 2006; Sudmant et al., 2015). Statistical analysis and plot visualizations were achieved using R scripts. The *t* test was performed to estimate the distributed significant difference in the CNV genotype between groups.

Pathway enrichment of candidate genes analysis (Kyoto Encyclopedia of Genes and Genomes) was displayed by a public database (http://kobas.cbi.pku.edu.cn/index.php) with a *p*‐value < .05. False discovery rate (FDR) correction was applied to the *p*‐values, with FDR ≤ 0.05 as a threshold (*Q*‐value).

## 3 RESULTS

After quality trimming, on average, approximately 97.4% (Q20) of the reads were retained from each individual. Approximately 98.1% of the HQR can be aligned to the goat reference genome (ARS1). After removing the polymerase chain reaction repeats, the average sequencing coverage of the 72 individuals was ~18.9× (average of ~25.7× for red‐bone goats and ~16.3× for common goats) as calculated by QualiMap v.2.2 (Okonechnikov et al., 2016). A total of 31,828,206 SNPs were identified for further selection pressure analysis.

**FIGURE 2:**
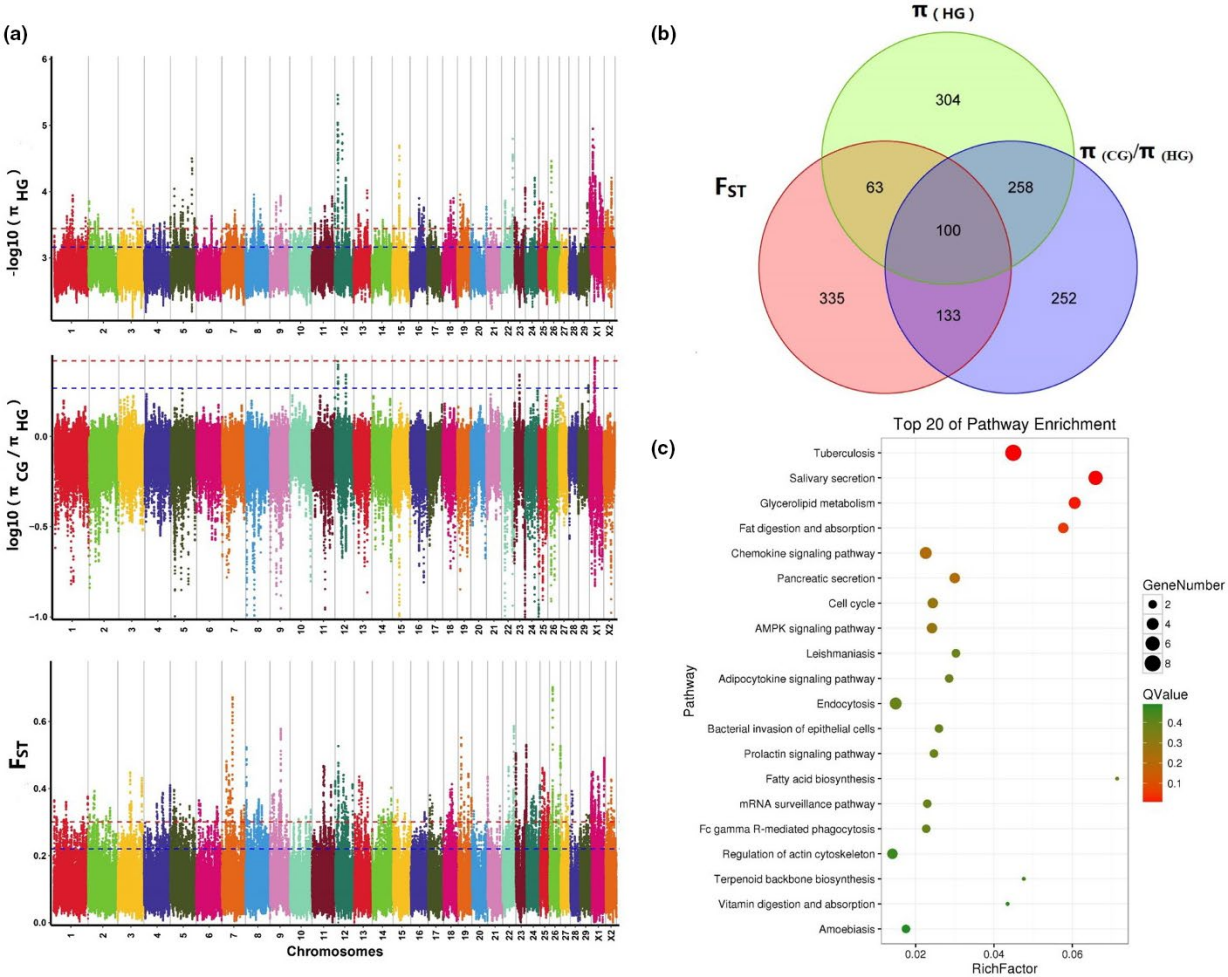
Genome‐wide selective sweeps of Mile red‐bone goats and annotation of candidate genes. (a) Genome‐wide selective sweep for the chromosome region in Mile red‐bone goats by using *π* (HG), *π* (CG)/*π* (HG), and *F*_ST_. Sliding window analysis (40 kb window with 25 kb step increment) used a 99‐percentile cutoff. (b) The 100 candidate genes obtained by interaction of *F*_ST_, *π* (HG), and *π* (CG)/*π* (HG). (c) Top 20 significantly enriched signaling pathways from candidate genes

Figure 2a shows the selection signature analysis results of all statistic parameters in 257,892 CWs with SNPs dataset. In total, 1889 CWs were assessed by top 1% *π* in HG (*π* < 0.000362), containing 725 genes. Furthermore, 776 CWs were assessed by top 1% of *π* (CG)/*π* (HG) (>2.614293), showing 743 genes. Lastly, 2586 windows were assessed using top 1% of *F*_ST_ (*F*_ST_ >0.300599), which contained 631 genes. A total of 100 candidate genes, such as *TMEM208*, *PNKD*, *GPBAR1*, *MAP4*, *SYNCRIP*, *ARFGEF1*, *FBXL8*, *CACNA2D2*, and *PNLIPRP*, were identified by combining the interaction from 1% windows with *π* (HG), *π* (CG)/*π*(HG), and *F*_ST_ (Figure 2b).

Functional classification and annotation of signaling pathways were performed for the top 100 genes. A total of 41 of the 100 genes were annotated in 77 molecular signaling pathways (top 20 enriched pathways in Figure 2c, Table S1). Of the 77 pathways, 25 were annotated in the organismal system, nine in metabolism, 19 in human disease, three in genetic information processing, six in cellular process, and 15 in environmental information processing classes. Nine (tuberculosis, salivary secretion, glycerolipid metabolism, fat digestion and absorption, chemokine signaling pathway, pancreatic secretion, cell cycle, AMP‐activated protein kinase signaling pathway, and Leishmaniasis) and three signal pathways (tuberculosis, salivary secretion, and glycerolipid metabolism) had significantly enriched *p*‐value and *Q*‐value, respectively.

**FIGURE 3:**
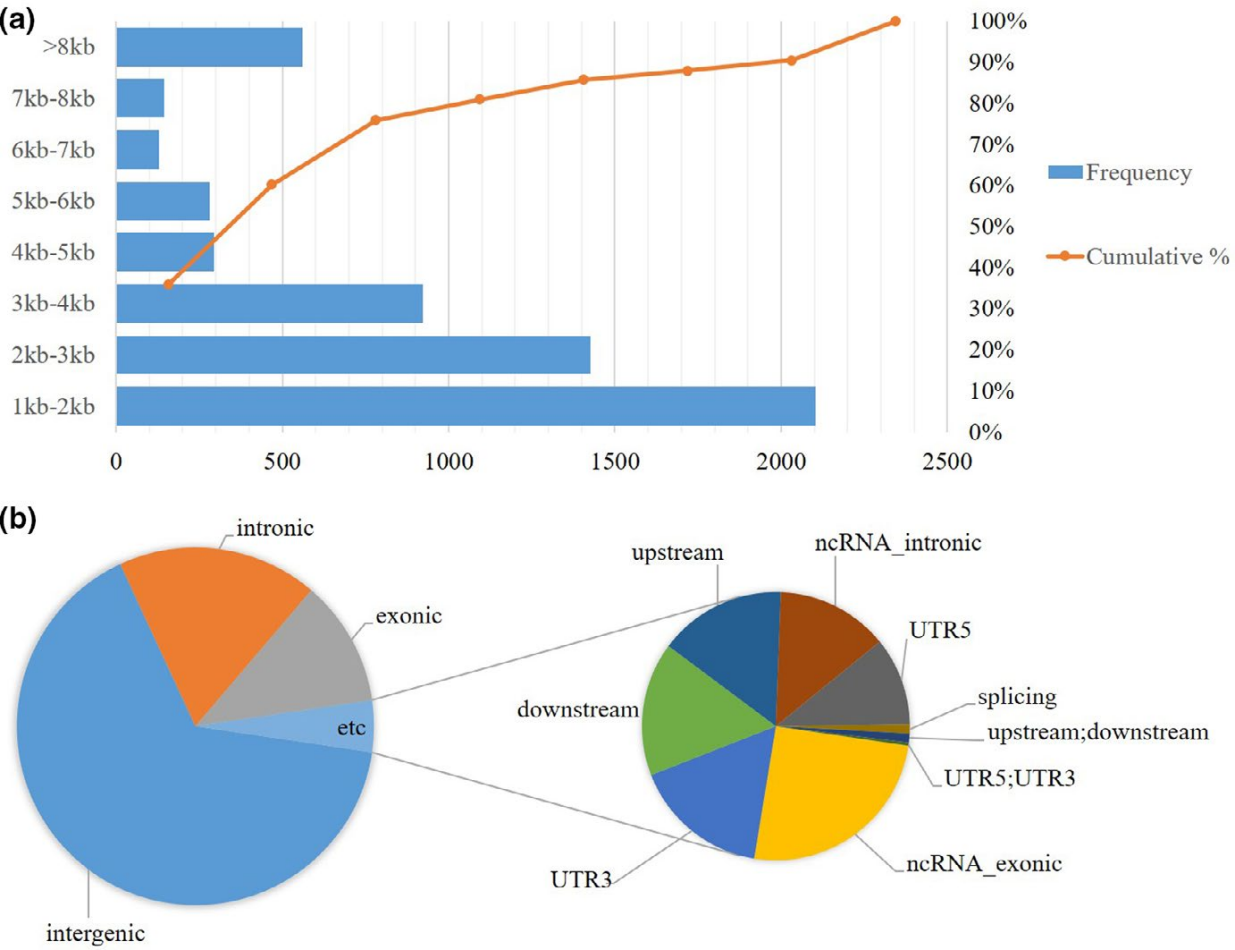
Length distribution and chromosome location annotation of CNVs. a: CNV size interval distribution; b: annotation type of chromosome location of CNVs

Subsequently, we detected 5862 CNV variants from the 72 goat individuals. The size of the CNVs identified varied from 1.59 kb to 1755.99 kb, with an average size of 6.82 kb and a median size of 2.39 kb (Figure 3a, Table S2). Figure 3b reveals the annotation type of CNVs.

**FIGURE 4:**
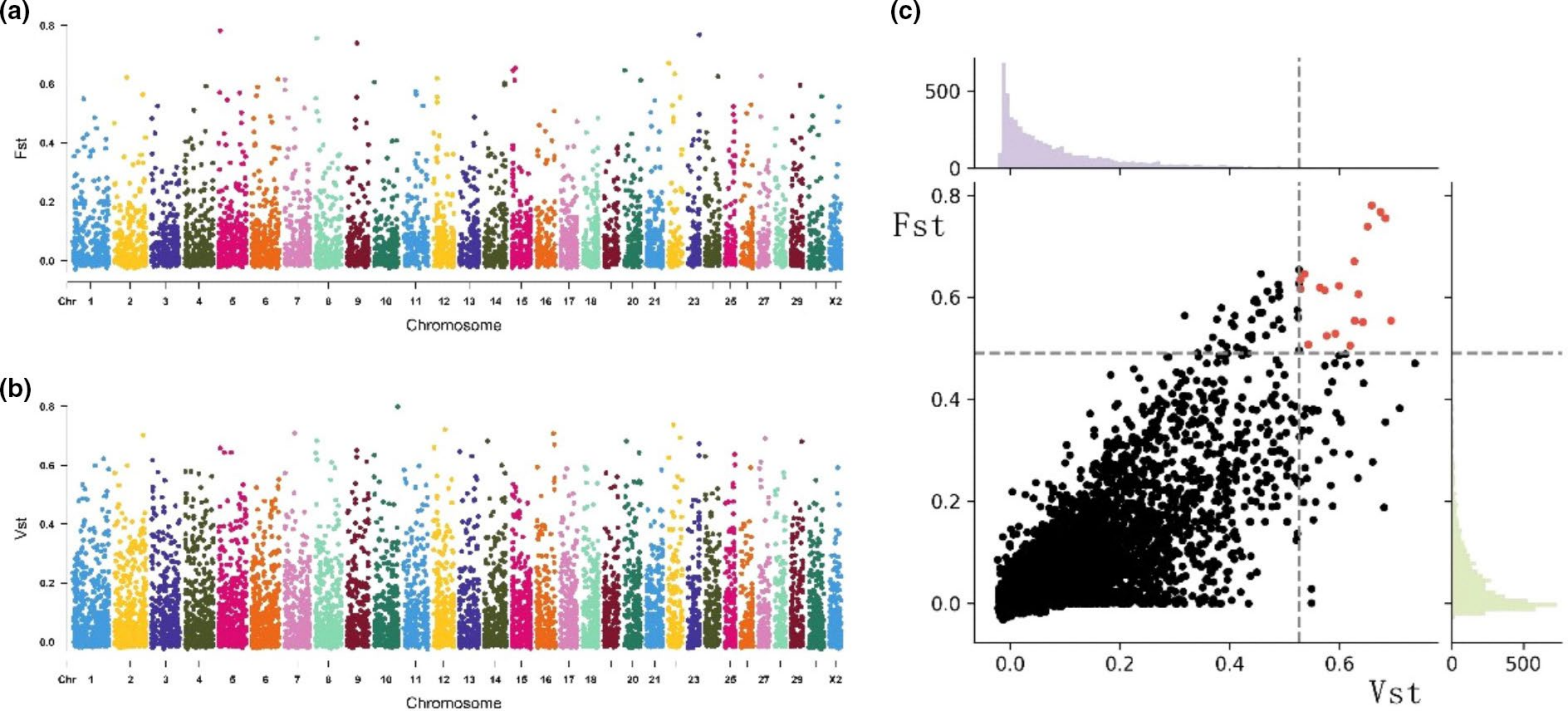
Genome‐wide selection scan for autosomal chromosome CNV in Mile red‐bone goats using *F*_ST_ and *V*_ST_. (a) Manhattan map of *F*_ST_; (b) Manhattan map of *V*_ST_, and (c) intersection of CNV between *F*_ST_ and *V*_ST_

According to the *F*_ST_ analysis of the CNV between the two groups, 57 CNVs were screened with the top 1% (*F*_ST_ > 0.490), which overlapped with 17 coding genes, such as *OSBPL8*, *GALNTL6*, *DOCK2*, *CAMK4*, *GLIPR1*, and *ARHGEF38* (Table S3, Figure 4a). Only *GALNTL6* and *CAMK4* were annotated in 13 pathways, such as OC differentiation and aldosterone synthesis and secretion (Table S4). Moreover, 59 CNVs were obtained with the top 1% (*V*_ST_ > 0.527) and covered 25 genes, such as *IFRD2*, *GALNTL6*, *PTDSS1*, and *MUC6* (Table S5, Figure 4b). Five genes were annotated in seven pathways (metabolic pathways, lysine degradation, glycerophospholipid metabolism, lysosome, endocytosis, neuroactive ligand–receptor interaction, and metabolic pathways) (Table S6).

Finally, 19 CNVs were identified based on the interaction of the top 1% CNVs with *F*_ST_ and *V*_ST_, and all those CNVs presented significant differences in the genotype frequency distribution between HG and CG (Table S7). Six CNVs overlapped with the intragenic (exonic/intronic) region of six coding genes (*IFRD2*, *GALNTL6*, *OSBPL8*, *SH3RF1*, *LOC108637255*, and *GFM2*). Furthermore, 29 genes (*IFRD2*, *GALNTL6*, *KHDRBS2*, *OSBPL8*, *TRNAS*‐*GGA*, *SH3RF1*, *TRNAH*‐*GUG*, *EGFR*, *GALNTL6*, *NONE*, *RNLS*, *EPS15*, *WDR36*, *LOC108637255*, *LOC102174324*, *LOC106501745*, *LDB2*, *CHL1*, *TRNAC*‐*ACA*, *TERB2*, *TRNAF*‐*GAA*, *SEC61G*, *TMEFF2*, *PTEN*, *OSBPL9*, *LOC102185832*, *NR5A2*, *QDPR*, and *CNTN3*) were annotated on the up‐ and downstream of CNVs with a 2 Mb range (Table S8), and one gene (*GALNTL6*) was annotated in mucin‐type O‐glycan biosynthesis. Metabolic pathways were also investigated (Table S9).

## 4 DISCUSSION

*Rubia cordifolia* is a plant that produces a natural dye, and pseudopurpurin is one of its components. Pseudopurpurin was first extracted from the roots of wild *R. cordifolia* by Hill (1934). Pseudopurpurin is metabolized out of the body (Richter, 1937) by combining with glucose to form glycosides after ingestion in animals. Wu, Wang, et al. (2012) observed that the substance causing the discoloration of bones in red‐bone goats is pseudopurpurin, which has a specific chemical affinity for calcium, thereby leading to increased bone mineral density. Therefore, we believe that the red‐bone phenotype of Mile goat is probably due to the madder consumption of grazing sheep, causing pseudopurpurin in the madder to gradually deposit in and stain the bone. Although *R. cordifolia* is extensively used as a source of herbivore feed worldwide (Cooksey, 2020), the physiological phenotype of red bone has not been extensively explored. The reason may be the selectivity of herbivores to plants or simple natural selection and long‐term evolution. Animal phenotypes and unique physiological phenomena originate from long‐term natural adaption and artificial selection (Dominguez‐Bello et al., 2019; Young & Hopkinson, 2017) and the specificity of genetic material, which helps promote the coevolution of animal and environment (Frantzeskakis et al., 2020).

In the present study, we identified a series of interesting genes and signaling pathways related to the physiological phenotype and high bone mineral density of red‐bone goat. Nicotinamide nucleotide adenylyltransferase 2 (*NMNAT2*) and pancreatic lipase (*PNLIP*) were annotated in vitamin metabolism‐related signaling pathways through SNP selective‐signal analysis. First, a previous study has shown that *PNLIP* is directly involved in the biosynthesis of vitamin A. Vitamin A affects the osteogenic stage differently by enhancing the differentiation of early osteoblasts and inhibiting bone mineralization through retinoic acid receptor signal transduction and the regulation of osteoblast/osteoblast‐related osteopeptide (Yee et al., 2021). Second, *NMNAT2* has been implicated in the biosynthesis of vitamin B, and nicotinamide mononucleotide is the most direct precursor of NAD^+^. Huang et al. demonstrated that NAD^+^ participates in the differentiation and maintenance of osteoblasts (Huang & Tao, 2020). These findings suggest that *NMNAT2* may indirectly participate in the regulation of bone differentiation through the precursor of NAD^+^. Meanwhile, osteoblasts are important cells for bone formation, development, and growth. They produce collagen fibers and matrix around bones and promote matrix calcification (Breitbart et al., 2000). Therefore, we speculated that these two genes may participate in the development of the special bone structure of Mile red‐bone goat by regulating the vitamin metabolism signaling pathway.

Calcium/calmodulin‐dependent protein kinase IV (*CAMK4*) and epidermal growth factor receptor (*EGFR*) genes were identified from the CNV selective‐signal analysis in this study. *CAMK4* is involved in the biological process of OC bone resorption (Wang et al., 2001) and is an important part of the calcium signaling pathway. Particularly, *CAMK4* is involved in the pathway mediated by the transcription factor cAMP response element binding and nuclear factor activated T, leading to OC differentiation (Jia et al., 2020).

Additionally, *EGFR* is annotated to the parathyroid hormone synthesis pathway, oxytocin signaling pathway, and calcium signaling pathway, which have been confirmed to be related to bone formation and differentiation (Ding et al., 2020; Kermgard et al., 2021). Linder et al. (2018) observed that mice with *EGFR* functional deficiency have osteoporosis, cartilage, and intramembrane ossification damage and irregular bone mineralization. Other studies suggested that *EGFR* is involved in bone formation through the regulation of bone morphogenetic proteins (BMPs) family genes (Bach et al., 2018).

During bone development, OCs attach onto the growth area of bone and release lactic acid and citric acid. Under the action of acids, calcium ions are broken down from the bone into the blood (Zhu et al., 2019). Pseudopurpurin reportedly has a special chemical affinity for calcium (Wu, Wang, et al., 2012) and can combine with calcium ions to form insoluble red calcium‐salt complexes (Hübner & Ic, 2010). Furthermore, the continuous expression of osteoblasts induces insoluble red calcium‐salt complexes contacting with the organic components of developing bones and making the newly formed bones turn red (Hübner & Ic, 2010). The identification of a series of calcium‐related metabolic signaling pathway genes in this study suggested that they may relate to the genetic basis of bone color in Mile red‐bone goat.

Accumulated pseudopurpurin easily combines with free calcium ions in the gastrointestinal tract to form a red calcium pseudopurpurin complex in vivo. This complex is popularly applied in the pathological‐section staining of bone tissue due to its adhesiveness (Muir, 1960). Therefore, when the calcium–pseudopurpurin complex makes contact with the organic components of developing bone (Richter, 1937), calcium ions are not lost during bone metabolism and continuously participate in the growth and development of bone, thereby forming the red‐bone character.

In the present study, we discovered another three candidate genes, namely, β‐1,4‐galactosyltransferase 6 (*B4GALT6*), galactosyltransferase 6 (*GALNTL6*), and quinoid dihydropteridine reductase (*QDPR*), related to metabolism. The function of *B4GALT6* is to catalyze the synthesis of lactose ceramide (Kwak et al., 2011) by transferring UDP galactose to glucosyl ceramide (GlcCer). *B4GALT6* catalyzes the combination of glucose and sphingomyelin to form glucocerebroside, which reduces the content of glucose in the body (Ramasamy et al., 2005). The expression of *B4GALT6* encoded by the *B4GALT6* gene reportedly affects glucose metabolism by reducing the efficiency of glucose bound to pseudopurpurin and promoting the deposition of pseudopurpurin in vivo. Our study confirmed that *GALNTL6* is annotated in the biosynthesis of mucin‐type O‐glycan. On one hand, this pathway contributes to the development of various tissues including bones (Tran & Ten Hagen, 2013). On the other hand, the O‐glycosylation mediated by *GALNT6* can promote the digestion of glucose by animal intestinal flora (Ramírez et al., 2020). Therefore, *GALNTL6* may also participate in the occurrence of the unique physiological bone phenotype of Mile red‐bone goats by regulating the glucose content in vivo.

The *QDPR* gene encodes dihydropteridine reductase in the biopterin metabolism pathway, which catalyzes the quinone dihydrobiopterin reduction mediated by NADH (Chandrashekaran et al., 2018; Si et al., 2017). *QDPR* gene is involved in hyperphenylalaninemia and has no direct relationship with glucose metabolism (Gundorova et al., 2021). However, the *QDPR* gene can indirectly regulate glucose levels (Xu et al., 2014) through folic acid. Li et al. observed that folic acid can reduce blood glucose level and increase insulin sensitivity (Wei et al., 2018). This finding suggested that the *QDPR* gene may indirectly affect folic acid content by encoding dihydropterin reductase, resulting in decreased glucose level. Therefore, these genes (*B4GALT6*, *GALNTL6*, and *QDPR*) may accelerate the metabolism of glucose in vivo and obstruct the process of glycoside formation by pseudopurpurin, resulting in metabolic blockage.

## 5 CONCLUSIONS

In this study, we performed genome‐wide selective scanning analysis and identified a series of candidate genes related to bone development and glucose metabolism. The findings are not only helpful to understanding the genetic basis of the unique red‐bone phenotype and structure of mile red‐bone goat but also to explaining the environmental and genetic factors that jointly affect the animal physiological phenotype.

## CONFLICT OF INTEREST

The authors declare no conflict of interest.

## AUTHOR CONTRIBUTIONS

**Yong‐Meng He:** Writing‐original draft (equal). **Qiong‐Hua Hong:** Resources (equal). **Dong‐Ke Zhou:** Formal analysis (supporting). **Shi‐Zhi Wang:** Formal analysis (supporting). **Bai‐Gao Yang:** Software (supporting). **Ying Yuan:** Formal analysis (supporting). **Wei‐Yi Zhang:** Formal analysis (supporting). **Yong‐Fu Huang:** Methodology (supporting). **Guang‐Xin E:** Methodology (equal); Writing‐review & editing (equal).

## INSTITUTIONAL REVIEW BOARD STATEMENT

The experimental conditions of this study were approved by the Committee on the Ethics of Animal Experiments of Southwest University (No. [2007] 3) and the Animal Protection Law of China.

## Supporting information

Supplementary Material

## Data Availability

Sequencing data generated in this study were deposited to the NCBI SRA database, with the accession number: SRR11696580–99.
